# Self‐Reported Hearing Impairment and Incident Frailty in English Community‐Dwelling Older Adults: A 4‐Year Follow‐Up Study

**DOI:** 10.1111/jgs.14687

**Published:** 2016-12-19

**Authors:** Ann E. M. Liljas, Livia A. Carvalho, Efstathios Papachristou, Cesar De Oliveira, S. Goya Wannamethee, Sheena E. Ramsay, Kate Walters

**Affiliations:** ^1^ Department of Primary Care and Population HealthUniversity College LondonLondonUnited Kingdom; ^2^ Department of Epidemiology and Public HealthUniversity College LondonLondonUnited Kingdom

**Keywords:** hearing impairment, frailty, older adults, aging

## Abstract

**Objectives:**

To examine the association between hearing impairment and incident frailty in older adults.

**Design:**

Cross‐sectional and longitudinal analyses with 4‐year follow‐up using data from the English Longitudinal Study of Ageing.

**Setting:**

Community.

**Participants:**

Community‐dwelling individuals aged 60 and older with data on hearing and frailty status (N = 2,836).

**Measurements:**

Hearing impairment was defined as poor self‐reported hearing. Having none of the five Fried frailty phenotype components (slow walking, weak grip, self‐reported exhaustion, weight loss and low physical activity) was defined as not frail, having one or two as prefrail, and having three or more as frail. Participants who were not frail at baseline were followed for incident prefrailty and frailty. Participants who were prefrail at baseline were followed for incident frailty.

**Results:**

One thousand three hundred ninety six (49%) participants were not frail, 1,178 (42%) were prefrail, and 262 (9%) were frail according to the Fried phenotype. At follow‐up, there were 367 new cases of prefrailty and frailty among those who were not frail at baseline (n = 1,396) and 133 new cases of frailty among those who were prefrail at baseline (n = 1,178). Cross‐sectional analysis showed an association between hearing impairment and frailty (age‐ and sex‐adjusted odds ratio (OR) = 1.66, 95% confidence interval (CI) = 1.37–2.01), which remained after further adjustments for wealth, education, cardiovascular disease, cognition, and depression. In longitudinal analyses, nonfrail participants with hearing impairment were at greater risk of becoming prefrail and frail at follow‐up (OR = 1.43, 95% CI = 1.05–1.95), but the association was attenuated after further adjustment. Prefrail participants with hearing impairment had a greater risk of becoming frail at follow‐up (OR = 1.64, 95% CI = 1.07–2.51) even after further adjustment.

**Conclusion:**

Hearing impairment in prefrail older adults was associated with greater risk of becoming frail, independent of covariates, suggesting that hearing impairment may hasten the progression of frailty.

Hearing impairment is common in later life and is estimated to affect 20% of adults in Great Britain aged 60 and older.^1^, ^2^ Age‐related hearing impairment has been associated with comorbidity, disability, and poor quality of life, affecting independent living and overall well‐being.^3^, ^4^ As the population ages,^5^ hearing impairment becomes an increasingly important public health concern. Another common health concern in later life is frailty.^6^, ^7^ Frailty is a clinical syndrome that refers to the body's inability to respond adequately to stressors because of multisystem impairments and lack of physiological reserve, increasing the risk of adverse outcomes including falls, hospitalization, institutionalization, and mortality.^6^, ^7^, ^8^, ^9^ Frailty is often described as a dynamic state along a continuum from normal aging to death,^10^ and transition between frailty states is common.^11^ Prefrailty is an intermediate stage between having no prevalent frailty and being frail,^7^ and an acute medical event or psychological stress that exceeds the individual's capacity for recovery often precipitates the transition from prefrail to frail.^11^, ^12^ One of the most commonly used frailty measures in the community setting is the Fried phenotype, which has been deemed suitable for identification of community‐dwelling older adults at risk of frailty and has been found to be associated with disability, mortality, and hospitalization.^7^, ^13^, ^14^ The Fried phenotype consists of five components: unintentional weight loss, weak grip, slow walking speed, exhaustion, and low levels of physical activity.^7^ A score of 0 refers to no frailty, 1 to 2 indicates prefrailty, and 3 or more is defined as frail. Research has shown that prefrail older adults have twice the risk of becoming frail as nonfrail older adults.^7^

Studies suggest that hearing impairment is associated with frailty^15^, ^16^ and its consequences, including functional decline^15^ and incident falls.^17^ A previous study found an association between self‐reported hearing impairment and frailty in older women but not men, but the study was cross‐sectional.^18^ The Health, Aging and Body Composition study found an association between hearing impairment and incident frailty at 10‐year follow‐up, but only two measures were used to define frailty (slow walking, inability to rise from a chair).^19^ None of these previous studies considered social interaction in the analyses, a factor associated with hearing impairment and frailty that might have mediated the associations observed.^20^ Moreover, little is known about the incidence of prefrailty and frailty. It is important to investigate the relationship between hearing impairment and frailty to determine whether prevention and treatment of hearing impairment in older age could minimize the burden of frailty on the individual and society. The current study examined the relationship between poor hearing and prefrailty and frailty in a nationally representative sample of community‐dwelling older people in England and the relationship between poor hearing and subsequent incident prefrailty and frailty over 4 years.

## Methods

### Study Design and Participants

This study used data from the English Longitudinal Study of Ageing (ELSA), a prospective study of a nationally representative sample of 11,392 men and women aged 50 and older who participated in the Health Survey for England in 1998, 1999, or 2001.^21^ The sample was drawn by postcode sector and stratified according to proportion of households in nonmanual socioeconomic groups and has been followed up every 4 years since 2004 with a nurse visit during which measurements of physical function were taken and an interview conducted on morbidity and lifestyle. For the purposes of this study, the study sample was restricted to participants with data on items in the Fried phenotype, including walking speed, which have only been measured in participants aged 60 and older. In 2004, interview and nurse data were obtained from 4,248 of 5,918 participants from the original study sample (core members) aged 60 and older. Complete data on hearing impairment, covariates, and frailty was obtained from 2,836 of these participants (67%) in 2004 and on frailty in 2008, who constituted the current study sample. Ethical approval was obtained from the Multicentre Research and Ethics Committee.

### Hearing Impairment

Hearing impairment was measured using a self‐reported, validated question previously demonstrated to be accurate when compared with objectively measured hearing^22^ asking participants to rate their hearing (using a hearing aid if they used one) as excellent, very good, good, fair, or poor. Reporting excellent, very good, or good hearing was classified as having good hearing, and this group formed the reference group. Experiencing fair or poor hearing was considered poor hearing.

### Assessment of Frailty

Frailty status was assessed in 2004, and participants were followed up for frailty from 2004 to 2008. Frailty was based on the five components of the Fried phenotype: weight loss, weak grip, slow walking, exhaustion, and low physical activity.^7^ These components were operationalized using definitions identical or very similar to those in the original phenotype studies.^7^, ^23^ Weight loss was defined as loss of 10% or more of body weight in the last 4 years or a current body mass index (BMI) of less than 18.5 kg/m^2^. Weak grip was assessed using a dynamometer with the maximum handgrip strength measure out of three attempts on each side used for analysis. Weak grip was classified as being in the lowest quintile of the sex‐ and BMI‐adjusted distribution. Slow walking speed was measured as the mean time of two measurements taken to complete an 8‐foot walk at usual pace. Height was divided at the sex‐specific median to categorize those in the lowest sex‐ and height‐specific quintile of the distribution as having slow walking speed. Participants in wheelchairs or who were bed bound, unable to walk because of health problems, or unable to walk alone were also counted as having slow walking. Exhaustion was defined as a positive response to either of the two statements “Felt that everything I did was an effort in the last week” or “Could not get going in the last week” from the Center for Epidemiologic Studies Depression Scale (CES‐D).^24^ Low physical activity was based on three questions about the frequency with which participants undertook vigorous, moderate, and mild exercise. Each question had four response options (more than once a week, once a week, one to three times a month, hardly ever or never) that were combined according to the amount and intensity of exercise involved. Reporting exercising hardly ever or never, mild exercise only, or moderate exercise a maximum of one to three times a month was classified as low physical activity. Frailty was defined as the presence of three or more of the five frailty components. Prefrailty was defined as the presence of one or two components. No prevalent frailty was defined as having none of the frailty components. The Fried phenotype was used because it incorporates a definition of prefrailty and frailty and is suitable for identification of community‐dwelling older adults at risk of frailty.^13^, ^14^

### Covariates

Age, sex, wealth, education, cardiovascular disease (CVD), smoking, hypertension, diabetes mellitus, history of falls, cognitive function, depression, and lack of companionship were considered as covariates. Age was grouped into 60 to 69, 70 to 79, and 80 and older. Total net nonpension wealth (financial, housing, physical wealth) of the household is presented according to quintile. Education was defined as having an intermediate or higher qualification versus no qualification. CVD has previously been associated with hearing impairment and frailty, so doctor‐diagnosed CVD (myocardial infarction, angina pectoris, stroke) and doctor‐diagnosed CVD‐related comorbidities including diabetes mellitus and hypertension were considered as potential covariates and analyzed dichotomously.^25^ Participants who reported that they had fallen in the last 12 months were classified as having had a fall. Smoking was defined as reporting being a current smoker and analyzed dichotomously. Cognitive function was measured using a validated 24‐point cognitive scale on time orientation and immediate and delay recall and analyzed continuously.^26^ The recall tests included a list of 10 words presented to participants, who were asked to recall as many words as possible immediately after the list was read and then again after an approximately 5‐minute delay during which they completed other survey questions. Questions on orientation to the day, date, month, and year were obtained from the Mini‐Mental State Examination (MMSE) and assessed by asking participants to report the day, date, month, and year.^27^, ^28^ Factors that may be on the causal pathway of hearing impairment and frailty were also considered, including depression and lack of companionship. Depression symptoms were based on the six questions on mood from the validated eight‐item version of the CES‐D, excluding the two questions on exhaustion that form part of the frailty phenotype definition.^24^ Scoring positively on two or more items indicated having depression symptoms and was analyzed dichotomously. Lack of companionship some of the time or often were combined and compared with no lack of companionship.

### Statistical Analyses

Logistic regression was used to assess the cross‐sectional relationships between hearing impairment and prefrailty and frailty combined. Logistic regression was also used to determine relationships between hearing impairment and incident prefrailty in participants without prevalent frailty at baseline between hearing impairment and incident frailty in participants with prefrailty at baseline followed for 4 years. Incident prefrailty and frailty in those without frailty at baseline were combined because of the small number of new frailty cases in this group (n = 24). Odds ratios (ORs) with 95% confidence intervals (CIs) were obtained using good hearing as the reference. Age, sex, and covariates that were significantly associated with hearing impairment in the nonfrail (Table 2) and prefrail groups (Table 3) (wealth, education, CVD, cognitive function) and that have consistently been associated with hearing impairment and frailty in previous studies, such as depression, were included in the statistical analyses.^29^, ^30^ If a positive association was demonstrated between hearing impairment and incident frailty, supplementary analyses were conducted to explore whether lack of companionship as a marker of social isolation and as a potential mechanism underlying the causal pathway between poor self‐reported hearing and frailty might explain this.^31^ All analyses were performed using SPSS version 22 (IBM Corp., Armonk, NY).

## Results

In 2004, 2,836 adults aged 60 and older completed an interview and underwent a nurse assessment. The prevalence of hearing impairment was 23% (n = 643), and 56% (n = 1584) of participants were men. One thousand three hundred ninety‐six participants (49%) were not frail, 1,178 (42%) were prefrail, and 262 (9%) were frail at baseline. At 4‐year follow‐up, there were 367 new cases of prefrailty (n = 343) and frailty (n = 24) among those who were not frail at baseline and 133 new cases of frailty among those who were prefrail at baseline.

### Cross‐sectional Associations of Hearing Impairment and Frailty at Baseline

Table 1 shows the characteristics of all participants and prevalence of frailty at baseline (2004) according to hearing impairment. Participants with poor hearing were more likely to be older, male, and less wealthy; have no educational qualification; have been diagnosed with CVD or diabetes mellitus; and have poorer cognitive function, symptoms of depression, and a history of falls than those with good hearing. Of older adults with poor hearing, 45% (n = 291) were prefrail, and 14% (n = 87) were frail, and of those with good hearing, 40% (n = 887) were prefrail, and 8% (n = 175) were frail.

**Table 1:** Age, Sex, Socioeconomic and Lifestyle Characteristics, Comorbidities, Falls, and Frailty Status in a Cohort of English Men and Women Aged 60 and Older in 2004 (Baseline)

Covariate	Overall, n = 2,836	Good Hearing, n = 2,193 (77%)	Poor Hearing, n = 643 (23%)	*P*‐Value
Age, n (%)				
60–69	1,526 (54)	1,266 (58)	260 (40)	<.001
70–79	1012 (36)	726 (33)	286 (45)	
≥80	298 (11)	201 (9)	97 (15)	
Male, n (%)	1,584 (56)	894 (41)	358 (56)	<.001
Wealth quintile, n (%)				
1 (lowest)	396 (14)	290 (13)	106 (17)	<.001
2	541 (19)	383 (18)	158 (25)	
3	562 (20)	430 (20)	132 (21)	
4	615 (22)	499 (23)	116 (18)	
5 (highest)	690 (24)	567 (26)	123 (19)	
No educational qualification, n (%)	1,086 ± 38	797 ± 36	289 ± 45	<.001
Smoker, n (%)	308 (11)	226 (10)	82 (13)	.08
Body mass index, kg/m^2^, mean ± SD	27.8 (4.6)	27.8 (4.6)	27.8 (4.6)	.67
Hypertension, n (%)	1,302 (46)	984 (45)	318 (50)	.40
Cardiovascular disease, n (%)	499 (18)	348 (16)	151 (24)	<.001
Diabetes mellitus, n (%)	251 (9)	175 (8)	76 (12)	<.001
Cognitive function, mean ± SD^a^	13.7 ± 3.3	14.0 ± 3.2	12.9 ± 3.4	<.001
Depression symptoms, n (%)	653 (23)	480 (22)	173 (27)	<.001
History of falls, n (%)	882 (31)	657 (30)	225 (35)	.02
Frailty status, n (%)				
Nonfrail	1,396 (49)	1,131 (52)	265 (41)	<.001
Prefrail	1,178 (42)	887 (40)	291 (45)	<.001
Frail	262 (9)	175 (8)	87 (14)	<.001

a According to a 24‐point cognitive scale on orientation to time and immediate and delayed recall.^26^

SD = standard deviation.

Cross‐sectional analyses of the relationship between frailty and hearing impairment showed that participants who were prefrail or frail were more likely to have poor hearing (age‐ and sex‐adjusted OR = 1.66, 95% CI = 1.37–2.01), and the association remained after further adjustment for wealth, education, CVD, cognition, and depression (OR = 1.41, 95% CI = 1.14–1.73). The relationship was further examined in a subsample of 2,663 participants with data on lack of companionship, and the association remained after additional adjustment for lack of companionship (OR = 1.44, 95% CI = 1.16–1.79). Additional analyses of the relationships between hearing impairment and prefrailty and frailty separately showed that frailty (OR = 1.52, 95% CI = 1.25–1.86) and prefrailty (OR = 2.32, 95% CI = 1.67–3.24) were significantly associated with hearing impairment. An interaction between hearing impairment and sex was tested for, and no association was found.

### Longitudinal Associations Between Hearing Impairment and Incident Prefrailty and Frailty

Two cohorts were constructed for longitudinal analyses—not frail at baseline and prefrail at baseline. Table 2 shows the characteristics of participants in the first cohort who were not frail at baseline according to hearing impairment. In this cohort, self‐reported hearing impairment was statistically significantly associated with older age, male sex, less wealth, no educational qualification, and poorer cognitive function. Symptoms of depression; history of falls; CVD; and CVD risk factors including smoking, BMI, hypertension, and diabetes mellitus were not associated with hearing impairment. Table 3 presents the characteristics of participants in the second cohort, who were prefrail at baseline, according to hearing impairment. In those who were prefrail at baseline, hearing impairment was associated with older age, male sex, CVD, and poorer cognitive function. No significant associations were observed between hearing impairment and wealth, education, smoking, BMI, hypertension, diabetes mellitus, symptoms of depression, or falls.

**Table 2:** Age, Sex, Socioeconomic and Lifestyle Characteristics, Comorbidities, and Falls in a Cohort of English Men and Women Aged 60 and Older with No Frailty in 2004

Covariate	Overall, n = 1,396	Good Hearing, n = 1,131 (81%)	Poor Hearing, n = 265 (19%)	*P*‐Value
Age, n (%)				
60–69	864 (62)	743 (66)	121 (46)	<.001
70–79	459 (33)	336 (30)	123 (46)	
≥80	73 (5)	52 (5)	21 (8)	
Male, n (%)	785 (56)	603 (53)	182 (69)	<.001
Wealth quintile, n (%)				
1 (lowest)	112 (8)	83 (7)	29 (11)	.01
2	204 (15)	154 (14)	50 (19)	
3	278 (20)	218 (20)	60 (23)	
4	346 (25)	293 (26)	53 (20)	
5 (highest)	436 (31)	368 (33)	68 (26)	
No educational qualification, n (%)	420 (30)	321 (28)	99 (37)	.04
Smoker, n (%)	103 (7)	82 (7)	21 (8)	.69
Body mass index, kg/m^2^, mean ± SD	27.2 ± 3.9	27.2 ± 3.9	27.3 ± 4.1	.85
Hypertension, n (%)	560 (40)	450 (40)	110 (42)	.61
Cardiovascular disease, n (%)	195 (14)	150 (13)	45 (17)	.12
Diabetes mellitus, n (%)	90 (6)	70 (6)	20 (8)	.42
Cognitive function, mean ± SD^a^	14.2 ± 3.3	14.4 ± 3.1	13.6 ± 3.2	<.001
Depression symptoms, n (%)	149 (11)	117 (10)	32 (12)	.41
History of falls, n (%)	347 (25)	278 (25)	69 (26)	.62

a According to a 24‐point cognitive scale on orientation to time and immediate and delayed recall.^26^

SD = standard deviation.

**Table 3:** Age, Sex, Socioeconomic and Lifestyle Characteristics, Comorbidities, and Falls in a Cohort of English Men and Women Aged 60 and Older with Prefrailty in 2004

Covariate	Overall, n = 1,178	Good Hearing, n = 887 (75%)	Poor Hearing, n = 291 (25%)	*P*‐Value
Age, n (%)				
60–69	568 (48)	458 (52)	110 (38)	<.001
70–79	455 (39)	327 (37)	128 (44)	
≥80	155 (13)	102 (12)	53 (18)	
Male, n (%)	413 (35)	261 (29)	152 (52)	<.001
Wealth quintile, n (%)				
1 (lowest)	195 (17)	144 (16)	51 (18)	.28
2	264 (22)	189 (22)	75 (26)	
3	242 (21)	182 (21)	60 (21)	
4	236 (20)	180 (21)	56 (19)	
5 (highest)	230 (20)	184 (21)	46 (16)	
No educational qualification, n (%)	507 (43)	372 (42)	135 (46)	.18
Smoker, n (%)	162 (14)	116 (13)	46 (16)	.24
Body mass index, kg/m^2^, mean ± SD	27.9 ± 4.8	28.0 ± 4.9	27.8 ± 4.4	.48
Hypertension, n (%)	583 (50)	430 (49)	153 (53)	.23
Cardiovascular disease, n (%)	213 (18)	144 (16)	69 (24)	<.001
Diabetes mellitus, n (%)	121 (10)	86 (10)	35 (12)	.26
Cognitive function, mean ± SD^a^	13.7 ± 3.4	13.8 ± 3.3	12.5 ± 3.4	<.001
Depression symptoms, n (%)	362 (31)	266 (30)	96 (33)	.32
History of falls, n (%)	396 (34)	291 (33)	105 (36)	.31

a According to a 24‐point cognitive scale on orientation to time and immediate and delayed recall.^26^

SD = standard deviation.

Table 4 presents the findings of incident prefrailty and frailty in participants who were not frail at baseline and of incident frailty in those who were prefrail at baseline. Of participants who were not frail at baseline, those with poor hearing were at greater risk of being prefrail or frail at 4‐year follow‐up (age‐ and sex‐adjusted OR = 1.43, 95% CI = 1.05–1.95) than those with good self‐reported hearing. The association was attenuated after further adjustment for wealth and education. Of participants who were prefrail at baseline, those who reported poor hearing were at greater risk of developing frailty than those with good hearing (age‐ and sex‐adjusted OR = 1.64, 95% CI = 1.07–2.51). This association remained after further adjustment for wealth, education, CVD, cognition, and depression. The association was further examined in a subsample of 1,088 prefrail participants with data on lack of companionship followed up for incident frailty over 4 years. Poor hearing remained associated with greater risk of frailty (OR = 1.72, 95% CI = 1.08–2.76) after further adjustment for lack of companionship.

**Table 4:** Associations Between Incidence of Prefrailty and Frailty and Hearing Impairment in English Men and Women Aged 60 and Older in 2004 Followed for 4 Years to 2008

Models for Adjustment	Odds Ratio (95% Confidence Interval) for Poor Hearing
No prevalent frailty at baseline^a^	
Model 1 (M1): age and sex	1.43 (1.05–1.95)
Model 2 (M2): M1 + wealth and education	1.32 (0.96–1.82)
Model 3 (M3): M2 + CVD	1.32 (0.96–1.82)
Model 4 (M4): M3 + cognition	1.31 (0.95–1.80)
Model 5 (M5): M4 + depression	1.32 (0.96–1.81)
Prefrail at baseline^b^	
Model 1 (M1): age and sex	1.64 (1.07–2.51)
Model 2 (M2): M1 + wealth and education	1.63 (1.06–2.52)
Model 3 (M3): M2 + CVD	1.62 (1.05–2.51)
Model 4 (M4): M3 + cognition	1.58 (1.02–2.45)
Model 5 (M5): M4 + depression	1.57 (1.01–2.44)

a Followed up for prefrailty and frailty. Good hearing, n = 1,131 (prefrail or frail at follow‐up, n = 280 (25%)); poor hearing, n = 265 (prefrail or frail at follow‐up, n = 87 (33%)).

b Followed‐up for frailty. Good hearing, n = 887 (frail at follow‐up, n = 91 (10%)); poor hearing, n = 291 (frail at follow‐up, n = 42 (14%)).

CVD = cardiovascular disease.

## Discussion

This study investigated the association between hearing impairment and incident frailty in older adults. The results show that prefrail older adults with self‐reported poor hearing are at greater risk of becoming frail than prefrail older adults with good hearing. The association observed remained after adjustment for covariates. To the best of the authors’ knowledge, this is the first study to investigate hearing impairment and incident frailty using the Fried phenotype.

In the longitudinal analyses, poor self‐reported hearing was significantly associated with greater risk of becoming prefrail and frail, although adjustment for markers of socioeconomic position (wealth and education) attenuated this. Lower socioeconomic position has consistently been associated with greater risk of frailty, an association largely explained by chronic diseases, depression, psychosocial factors, and lower income.^32^, ^33^, ^34^ Manual social class, chronic diseases, and psychosocial factors have also been associated with hearing impairment,^2^, ^4^ which may explain why the association observed was not independent of socioeconomic status. In contrast, hearing impairment was associated with greater risk of developing frailty in prefrail older adults, and the association remained after further adjustment for covariates. This finding is consistent with those of a previous longitudinal study showing an association between hearing impairment and difficulty getting out of chair and slow gait speed,^19^ suggesting that hearing impairment may increase the risk of future frailty in older adults already showing signs of frailty. An important aspect of the current study results is that the association between hearing impairment and incident frailty was observed in individuals who were prefrail but not in those who were not frail at baseline. Poor hearing may therefore be a particular problem in older adults who have started to become frail, suggesting that hearing impairment hastens the progression of frailty. This supports the cumulative deficit model of frailty,^35^ suggesting that poor subjective hearing adds to other accumulating health deficits in increasing the risk of frailty. These findings suggest that hearing impairment may be an important deficit to consider.

In the cross‐sectional analyses, hearing impairment was associated with prefrailty and frailty separately and combined. The longitudinal finding confirms the directionality of the association observed in the cross‐sectional analysis and supports the possibility that hearing impairment may increase the risk of frailty, although several factors could explain the association observed in the longitudinal study. For example, it has been hypothesized that social support may help to minimize the effect of loss of physiological reserve associated with frailty.^20^ Nevertheless, communication problems due to hearing impairment may restrict social engagement and therefore access to social support.^31^ Consequently, prefrail individuals with hearing impairment may not be able to benefit from the positive effects of social support in preventing further decline. Unmeasured comorbidities such as anxiety^36^ or comorbidities that were poorly assessed in the ELSA, including self‐reported doctor‐diagnosed CVD and limited aspects of cognition (further discussed below), conditions associated with hearing impairment and frailty^4^, ^7^, ^37^ may also explain the relationship. Finally, the association could be due to shared pathological pathways such as inflammation, which is related to hearing impairment and frailty.^38^, ^39^

### Strengths and Limitations

The major strengths of this study are that it was a large sample taken from a representative cohort of the community‐dwelling English population aged 60 and older.^40^ A prospective study design was used, participants were followed for 4 years for prefrailty and frailty, and the models were adjusted for several important potential confounding factors. Limitations include that hearing impairment was self‐reported rather than objectively measured, although the question used has been validated against objective measures, and hearing prevalence was comparable with national estimates and previous studies using objective measures.^1^, ^4^, ^16^, ^41^ Hearing depends on good cognitive function.^37^, ^42^ Cognitive function based on orientation and immediate and delayed recall was adjusted for, although a limitation of this study was that other aspects of cognitive function or measures of dementia were not available. The investigations, therefore, did not fully account for the role of cognition or dementia in explaining the association between hearing impairment and frailty. Because of data limitations, a slightly modified version of the validated Fried phenotype was used to assess frailty. Although objectively measured data on weight loss over time were used, intentional and unintentional weight loss could not be differentiated, and levels of physical activity referred to frequency and intensity of exercise, without information on calorie consumption. Nevertheless, data were obtained through interviews and nurse assessments, and the findings on prevalence of frailty are comparable with those in the original Fried phenotype study.^7^ The current study was restricted to the two‐thirds of ELSA participants with data on frailty measurements at baseline and follow‐up. Nonrespondents tended to be older and in poorer health, suggesting that prevalence rates of hearing impairment, prefrailty, and frailty might have been higher in nonresponders.^40^ Data on hearing aid use were not available, restricting information on any preventable action that participants may have taken. Hearing impairment was measured only at baseline, and no information on the primary cause of and change in hearing impairment were investigated. Finally, ELSA includes people predominantly of white British ethnic origin, and generalization of findings to other ethnic groups is therefore limited.

### Implications

The finding that hearing impairment in prefrail older adults increases the risk of becoming frail is of public health importance because hearing impairment and frailty affect a large proportion of older adults.^1^, ^7^ Identifying and actively managing hearing impairment in prefrail older adults may have potential to delay development of frailty. Poor hearing might to some extent be corrected; a population‐based study showed that only 15% of older adults with objectively assessed hearing impairment use a hearing aid.^43^ Self‐reported hearing impairment may be remediable in that it can be addressed with hearing aids, cochlear implants, and interventions aiming to optimize social and environmental conditions for hearing.^44^, ^45^ The progression from prefrailty to frailty appears to be predominately an end‐stage, presaging death,^7^ although frailty is potentially preventable if targeted in the early stages^7^, ^11^, ^46^ through interventions such as physical exercise^47^, ^48^, ^49^ previously shown to be effective in prefrail individuals.^47^, ^48^ Avoiding frailty could reduce adverse health outcomes such as falls, hospitalization, institutionalization, and the associated financial costs. Addressing hearing problems in prefrail individuals may therefore be critical and has the potential to reduce the burden of poor health on individuals and society.

A second implication of these findings is that hearing impairment could be an important component of assessments aimed at identifying individuals at risk of frailty. Few assessments or indices such as the Frailty Index include hearing impairment.^50^ The current findings suggest that future research using such indices to identify those with potential frailty should consider including hearing impairment because it was found to be predictive of the change from prefrailty to frailty. Further research is warranted on the possible mechanisms of frailty in hearing‐impaired older adults.

## Conclusions

Self‐reported hearing impairment is associated with prefrailty and frailty in older age. Furthermore, prefrail older adults with poor hearing have a greater risk of becoming frail in the following 4 years, suggesting that hearing impairment may exacerbate the progression of frailty in individuals who are already prefrail.

## References

[1] Akeroyd MA , Foreman K , Holman JA . Estimates of the number of adults in England, Wales, and Scotland with a hearing loss. Int J Audiol 2014;53:60–61. 24274676 10.3109/14992027.2013.850539 PMC3894713

[2] Liljas AE , Wannamethee SG , Whincup PH et al. Socio‐demographic characteristics, lifestyle factors and burden of morbidity associated with self‐reported hearing and vision impairments in older British community‐dwelling men: A cross‐sectional study. J Public Health (Oxf) 2016;38:e21–e28. 26177816 10.1093/pubmed/fdv095

[3] Campbell VA , Crews JE , Moriarty DG et al. Surveillance for sensory impairment, activity limitation, and health‐related quality of life among older adults—United States, 1993–1997. MMWR CDC Surveill Summ 1999;48:131–156. 10634273

[4] Crews JE , Campbell VA . Vision impairment and hearing loss among community‐dwelling older Americans: Implications for health and functioning. Am J Public Health 2004;94:823–829. 15117707 10.2105/ajph.94.5.823 PMC1448344

[5] Office for National Statistics . National Population Projections, 2012‐based. Chapter 2, Summary results, 2014 [on‐line]. Available at www.ons.gov.uk/ons/dcp171776_355182.pdf Accessed April 17, 2016.

[6] Ng TP , Feng L , Nyunt MS et al. Frailty in older persons: Multisystem risk factors and the Frailty Risk Index (FRI). J Am Med Dir Assoc 2014;15:635–642. 24746590 10.1016/j.jamda.2014.03.008

[7] Fried LP , Tangen CM , Walston J et al. Frailty in older adults: Evidence for a phenotype. J Gerontol A Biol Sci Med Sci 2001;56A:M146–M156. 10.1093/gerona/56.3.m146 11253156

[8] Rockwood K , Stadnyk K , MacKnight C et al. A brief clinical instrument to classify frailty in elderly people. Lancet 1999;353:205–206. 9923878 10.1016/S0140-6736(98)04402-X

[9] Mitnitski A , Fallah N , Rockwood K . A multistate model of cognitive dynamics in relation to frailty in older adults. Ann Epidemiol 2011;21:507–516. 21435904 10.1016/j.annepidem.2011.01.006

[10] Abellan van Kan G , Rolland Y , Bergman H et al. The I.A.N.A Task Force on frailty assessment of older people in clinical practice. J Nutr Health Aging 2008;12:29–37. 18165842 10.1007/BF02982161 PMC12892609

[11] Gill TM , Gahbauer EA , Allore HG et al. Transitions between frailty states among community‐living older persons. Arch Intern Med 2006;166:418–423. 16505261 10.1001/archinte.166.4.418

[12] Lang PO , Michel JP , Zekry D . Frailty syndrome: A transitional state in a dynamic process. Gerontology 2009;55:539–549. 19346741 10.1159/000211949

[13] Bouillon K , Kivimaki M , Hamer M et al. Measures of frailty in population‐based studies: An overview. BMC Geriatr 2013;13:64. 23786540 10.1186/1471-2318-13-64 PMC3710231

[14] Rockwood K , Andrew M , Mitnitski A . A comparison of two approaches to measuring frailty in elderly people. J Gerontol A Biol Sci Med Sci 2007;62A:738–743. 10.1093/gerona/62.7.738 17634321

[15] Strawbridge WJ , Wallhagen MI , Shema SJ et al. Negative consequences of hearing impairment in old age: A longitudinal analysis. Gerontologist 2000;40:320–326. 10853526 10.1093/geront/40.3.320

[16] Dalton DS , Cruickshanks KJ , Klein BE et al. The impact of hearing loss on quality of life in older adults. Gerontologist 2003;43:661–668. 14570962 10.1093/geront/43.5.661

[17] Lin FR , Ferrucci L . Hearing loss and falls among older adults in the United States. Arch Intern Med 2012;172:369–371. 22371929 10.1001/archinternmed.2011.728 PMC3518403

[18] Kamil RJ , Li L , Lin FR . Association between hearing impairment and frailty in older adults. J Am Geriatr Soc 2014;62:1186–1188. 24925554 10.1111/jgs.12860 PMC4141776

[19] Kamil RJ , Betz J , Powers BB et al. Association of hearing impairment with incident frailty and falls in older adults. J Aging Health 2016;28:644–660. 26438083 10.1177/0898264315608730 PMC5644033

[20] Xue QL . The frailty syndrome: Definition and natural history. Clin Geriatr Med 2011;27:1–15. 21093718 10.1016/j.cger.2010.08.009 PMC3028599

[21] Marmot M , Oldfield Z , Clemens S et al. English Longitudinal Study of Ageing: Waves 0–6, 1998–2013, 23rd Ed London: UK Data Service, 2015.

[22] Ferrite S , Santana VS , Marshall SW . Validity of self‐reported hearing loss in adults: Performance of three single questions. Rev Saude Publica 2011;45:824–830. 10.1590/s0034-89102011005000050 21808834

[23] Bandeen‐Roche K , Xue QL , Ferrucci L et al. Phenotype of frailty: Characterization in the Women's Health and Aging Studies. J Gerontol A Biol Sci Med Sci 2006;61A:262–266. 10.1093/gerona/61.3.262 16567375

[24] Wallace RB , Herzog AR , Ofstedal MB et al. Documentation of Affective Functioning Measures in the Health and Retirement Study: Survey Research Center. Ann Arbor, MI: University of Michigan, 2000.

[25] Newman AB , Gottdiener JS , McBurnie MA et al. Associations of subclinical cardiovascular disease with frailty. J Gerontol A Biol Sci Med Sci 2001;56A:M158–M166. 10.1093/gerona/56.3.m158 11253157

[26] Langa KM , Llewellyn DJ , Lang IA et al. Cognitive health among older adults in the United States and in England. BMC Geriatr 2009;9:23. 19555494 10.1186/1471-2318-9-23 PMC2709651

[27] Lee S , Kawachi I , Berkman LF et al. Education, other socioeconomic indicators, and cognitive function. Am J Epidemiol 2003;157:712–720. 12697575 10.1093/aje/kwg042

[28] Weuve J , Kang JH , Manson JE et al. Physical activity, including walking, and cognitive function in older women. JAMA 2004;292:1454–1461. 15383516 10.1001/jama.292.12.1454

[29] Naramura H , Nakanishi N , Tatara K et al. Physical and mental correlates of hearing impairment in the elderly in Japan. Audiology 1999;38:24–29. 10052833 10.3109/00206099909072999

[30] Buigues C , Padilla‐Sanchez C , Garrido JF et al. The relationship between depression and frailty syndrome: A systematic review. Aging Ment Health 2015;19:762–772. 25319638 10.1080/13607863.2014.967174

[31] Weinstein BE , Ventry IM . Hearing impairment and social isolation in the elderly. J Speech Hear Res 1982;25:593–599. 7162161 10.1044/jshr.2504.593

[32] Hoogendijk EO , van Hout HP , Heymans MW et al. Explaining the association between educational level and frailty in older adults: Results from a 13‐year longitudinal study in the Netherlands. Ann Epidemiol 2014;24:538–544.e2. 24935466 10.1016/j.annepidem.2014.05.002

[33] Etman A , Kamphuis CB , van der Cammen TJ , et al. Do lifestyle, health and social participation mediate educational inequalities in frailty worsening? Eur J Public Health 2015;25:345–350. 25061232 10.1093/eurpub/cku093 PMC4447813

[34] Soler‐Vila H , Garcia‐Esquinas E , Leon‐Munoz LM et al. Contribution of health behaviours and clinical factors to socioeconomic differences in frailty among older adults. J Epidemiol Community Health 2016;70:354–360. 26567320 10.1136/jech-2015-206406

[35] Mitnitski AB , Mogilner AJ , Rockwood K . Accumulation of deficits as a proxy measure of aging. Sci World J 2001;1:323–336. 10.1100/tsw.2001.58 PMC6084020 12806071

[36] Mohlman J . Cognitive self‐consciousness—a predictor of increased anxiety following first‐time diagnosis of age‐related hearing loss. Aging Ment Health 2009;13:246–254. 19347691 10.1080/13607860802428026

[37] Amieva H , Ouvrard C , Giulioli C et al. Self‐reported hearing loss, hearing aids, and cognitive decline in elderly adults: A 25‐year study. J Am Geriatr Soc 2015;63:2099–2104. 26480972 10.1111/jgs.13649

[38] Collerton J , Martin‐Ruiz C , Davies K et al. Frailty and the role of inflammation, immunosenescence and cellular ageing in the very old: Cross‐sectional findings from the Newcastle 85 + Study. Mech Ageing Dev 2012;133:456–466. 22663935 10.1016/j.mad.2012.05.005

[39] Verschuur CA , Dowell A , Syddall HE et al. Markers of inflammatory status are associated with hearing threshold in older people: Findings from the Hertfordshire Ageing Study. Age Ageing 2012;41:92–97. 22086966 10.1093/ageing/afr140

[40] Banks J , Breeze E , Lessof C et al. Retirement, Health and Relationships of the Older Population in England. The 2004 English Longitudinal Study of Ageing (Wave 2). London: Institute for Fiscal Studies, 2006.

[41] Gibson WK , Cronin H , Kenny RA et al. Validation of the self‐reported hearing questions in the Irish Longitudinal Study on Ageing against the Whispered Voice Test. BMC Res Notes 2014;7:361. 24928453 10.1186/1756-0500-7-361 PMC4077556

[42] Arlinger S , Lunner T , Lyxell B et al. The emergence of cognitive hearing science. Scand J Psychol 2009;50:371–384. 19778385 10.1111/j.1467-9450.2009.00753.x

[43] Popelka MM , Cruickshanks KJ , Wiley TL et al. Low prevalence of hearing aid use among older adults with hearing loss: The Epidemiology of Hearing Loss Study. J Am Geriatr Soc 1998;46:1075–1078. 9736098 10.1111/j.1532-5415.1998.tb06643.x

[44] Lin FR , Metter EJ , O'Brien RJ et al. Hearing loss and incident dementia. Arch Neurol 2011;68:214–220. 21320988 10.1001/archneurol.2010.362 PMC3277836

[45] Zhan W , Cruickshanks KJ , Klein BE et al. Generational differences in the prevalence of hearing impairment in older adults. Am J Epidemiol 2010;171:260–266. 20008889 10.1093/aje/kwp370 PMC2878102

[46] Espinoza SE , Jung I , Hazuda H . Frailty transitions in the San Antonio Longitudinal Study of Aging. J Am Geriatr Soc 2012;60:652–660. 22316162 10.1111/j.1532-5415.2011.03882.x PMC3325321

[47] Gill TM , Baker DI , Gottschalk M et al. A program to prevent functional decline in physically frail, elderly persons who live at home. N Engl J Med 2002;347:1068–1074. 12362007 10.1056/NEJMoa020423

[48] Faber MJ , Bosscher RJ , Chin APMJ et al. Effects of exercise programs on falls and mobility in frail and pre‐frail older adults: A multicenter randomized controlled trial. Arch Phys Med Rehabil 2006;87:885–896. 16813773 10.1016/j.apmr.2006.04.005

[49] Fiatarone MA , O'Neill EF , Ryan ND et al. Exercise training and nutritional supplementation for physical frailty in very elderly people. N Engl J Med 1994;330:1769–1775. 8190152 10.1056/NEJM199406233302501

[50] Abellan van Kan G , Rolland Y , Houles M et al. The assessment of frailty in older adults. Clin Geriatr Med 2010;26:275–286. 20497846 10.1016/j.cger.2010.02.002

